# Preoperative Radiologic Classification of Convexity Meningioma to Predict the Survival and Aggressive Meningioma Behavior

**DOI:** 10.1371/journal.pone.0118908

**Published:** 2015-03-18

**Authors:** Yi Liu, Silky Chotai, Ming Chen, Shi Jin, Song-tao Qi, Jun Pan

**Affiliations:** Department of Neurosurgery, Nanfang Hospital of Southern Medical University, Guangzhou, China; The George Washington University, UNITED STATES

## Abstract

**Background:**

A subgroup of meningioma demonstrates clinical aggressive behavior. We set out to determine if the radiological parameters can predict histopathological aggressive meningioma, and propose a classification to predict survival and aggressive meningioma behavior.

**Methods:**

A retrospective review of medical records was conducted for patients who underwent surgical resection of their convexity meningioma. WHO-2007 grading was used for histopathological diagnosis. Preoperative radiologic parameters were analyzed, each parameter was scored 0 or 1. Signal intensity on diffusion weighted MRI (DWI) (hyperintensity=1), heterogeneity on T1-weighted gadolinium enhanced MRI (heterogeneity=1), disruption of arachnoid at brain-tumor interface=1and peritumoral edema (PTE) on T2-weighted MRI (presence of PTE=1) and tumor shape (irregular shape=1). Multivariate logistic regression analyses were conducted to determine association of radiological parameters to histopathological grading. Kaplan-Meier and Cox regression models were used to determine the association of scoring system to overall survival and progression free survival (PFS). Reliability of the classification was tested using Kappa co-efficient analysis.

**Results:**

Hyperintensity on DWI, disruption of arachnoid at brain-tumor interface, PTE, heterogenicitiy on T1-weighted enhanced MRI and irregular tumor shape were independent predictors of non-grade I meningioma. Mean follow-up period was 94.6 months (range, 12-117 months). Median survival and PFS in groups-I, II and III was 114.1±1.2 and 115.7± 0.8, 88± 3.3 and 58.5±3.9, 43.2± 5.1 and 18.2±1.7 months respectively. In cox regression analysis model, age (P<0.0001, OR–1.039, CI-1.017-0.062), WHO non-grade-I meningioma (P=0.017, OR–3.014, CI-1.217-7.465), radiological classification groups II (P=0.002, OR–6.194, CI–1.956-19.610) and III (P<0.0001, OR–21.658, CI–5.701-82.273) were independent predictors of unfavorable survival outcomes.

**Conclusions:**

Preoperative radiological classification can be used as a supplement to the histopathological grading. Group-I meningiomas demonstrate benign radiological, histopathological and clinical features; group-III demonstrates aggressive features. Group-II meningiomas demonstrate intermediate features; the need for more aggressive follow-up and/or treatment should be further investigated.

## Introduction

Meningiomas account for 20–32% of all the primary intracranial tumors[1–4]. According to the WHO 2007 classification system, the meningiomas are classified into 3 histological grades and 15 subtypes. This histopathological classification is generally used to predict the clinical course of meningioma. Most meningiomas are benign, well-circumscribed, slow growing tumors corresponding to WHO grade I[3] and usually follows uneventful clinical course. Some meningiomas, including WHO grade II (atypical) and grade III (anaplastic) tumors, are clinically and histologically aggressive. Grade II meningioma account for 4.7% to 7.2% and Grade III tumors comprises 1.0 to 2.8% of all the meningiomas[6–9]; however much larger proportion, 20% of the meningioma, demonstrates aggressive histological and/or clinical behavior[5]. This suggests that a borderline group of grade I meningioma also exists which behaves aggressively and might have recurrent or progressive disease[9]. Therefore, a histopathological grading alone might not accurately correlate with the patient outcome. It is important to distinguish WHO-grade I meningiomas with aggressive behavior from their non-aggressive counterparts. Several immunohistochemical parameters including Ki-67/ MIB-1, MMP-9, PR, ER are used as an adjunct to the histopathological grading to predict the meningioma prognosis.[4,10–13] Similarly, several radiological features are used in conjunction with histopathological grading to identify benign versus aggressive meningioma features. The loss of tumor-brain interface, presence of PTE, irregular tumor shape, heterogeneous enhancement on MRI, decreased apparent diffusion coefficient (ADC) in diffusion weighted imaging (DWI) and fluorodeoxyglucose F [8]PET predicts the aggressive histological and clinical behavior of meningioma [2,10,14–21,22–24,25,15].Despite of the numerous studies determining the clinical, radiological and histological parameters associated with aggressive meningioma behavior; the accurate prediction of meningioma behavior is challenging. We set out to determine if the radiological parameters can predict histopathological aggressive meningioma, and based on that propose a classification to predict survival and aggressive meningioma behavior.

## Material and Methods

After approval from the institutional review board, a retrospective review of the medical records, preoperative imaging and operative details was conducted for each patient. This retrospective study was approved by Nanfang Hospital Medical Ethics Committee. Patient records/information was anonymized and de-identified prior to analysis. The clinical records of participants in this study were de-identified prior to analysis.

### Patient demographics

Between 2003 and 2006, 246 patients with intracranial convexity meningiomas underwent surgery as the primary treatment at our institution. Patients underwent surgical resection without preoperative embolization. To nullify the effect of location (skull base versus convexity) [8,9,23],extent of resection[26,27]and preoperative functional status of the patients, we only included patients with convexity meningioma, Karnofsky performance score (KPS) of ≥60 and in whom Simpson grade I resection was achieved. Preoperative MRI, operative notes and surgical specimen were re-evaluated. The histopathology slides were re-evaluated and the histopathological diagnosis was classified based on the 2007 WHO classification system for meningioma[3].

### MR Imaging

MRI examinations were performed using a 1.5-T machine for patients operated on before 2004 and a 3-T machine for patients operated on after 2004 (General Electric Signa Excite HD). The MRI protocol included the following sequences: T1-weighted images (TR/TE, 436/21 msec), T2-weighted images (TR/TE, 5000/125 msec; echo train length 8), diffusion-weighted imaging (DWI, TR/TE,8000/65msec;Δ/δ37/32 msec) and FLAIR images (TR/TE/TI, 9000/145/2100 msec). Slice thickness was 5 mm, and the field of view varied between 18 and 30 cm. We also obtained axial, coronal, and sagittal T1-weighted images after administration of 0.1 mmol/kg of body weight of Gd-DTPA.

According to the WHO 2007 classification system, increased cellularity, necrosis and brain invasion are the histological features associated with non-Grade I tumor[3].Radiological appearance of these histological features has been described as hyperintensity on diffusion-weighted imaging (DWI), heterogeneous enhancement on T1-weighted gadolinium (Gd) enhanced MRI and cortical penetration or disappearance of arachnoid layer on T2-weighted MRI. Few studies have demonstrated that peritumoral edema (PTE) [2,8,15,28,29]and tumor shape [8,14,21,30] are associated with aggressive behavior or higher WHO grades. We evaluated the association of this five radiological parameters to the aggressive meningioma behavior; (1) signal intensity on DWI, (2) heterogeneity on T1-weighted Gd enhanced MRI, (3) Arachnoid layer on T2-weighted MRI, (4) PTE on T2-weighted MRI, (5) Tumor shape.

### Scoring of radiologic characteristics

**Fig 1:**
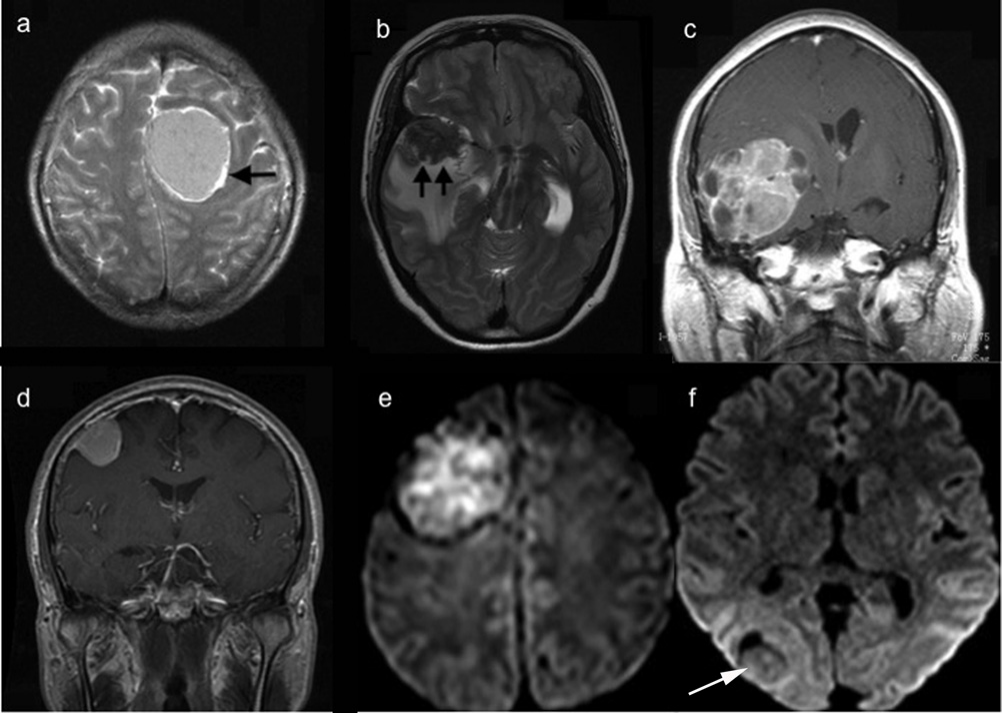
Radiologic characteristics of meningiomas in preoperative MRI. a) T2-weighted MRI demonstrating the complete arachnoid ring (black arrow) and no peritumoral edema; b) T2-weighted MRI demonstrating the disappearance of arachnoid ring (black arrow head) and peritumoral edema c) T1-weighted gadolinium enhanced MRI demonstrating heterogeneous enhancement and irregular shape of the tumor; d) T1-weighted gadolinium enhanced MRI demonstrating homogenous enhancement and regular shape of tumor; e) Diffusion weighted MRI demonstrating hyperintense lesion; f) Diffusion weighted MRI demonstrating isointense lesion.

Radiological features were scored as: DWI signal intensity (hyperintense to grey matter = 1,others = 0); T1-weighted Gd-enhanced MRI (heterogeneity = 1, homogeneity = 0); arachnoid layer on T2-weighted MRI (disappeared or disintegrated = 1, intact = 0); PTE on T2-weighted MRI (tumor with edema = 1, tumor without edema = 0) and tumor shape (tumor with irregular shape, including mushroom shape or lobulated = 1, tumor with regular shape, including globular shape = 0) (Fig. 1). The lowest score was 0 and the highest was 5 (Table 1). Preoperative MRI was evaluated and total score was calculated for each patient. Based on their preoperative MRI scoring, all patients were classified into 3 groups: group one = 0–1, group two = 2–3 and group three 4–5. The survival time, progression free survival and overall survival (OS) rates of each group were analyzed. The survival outcome was evaluated as favorable if the patients were alive at the last follow-up and unfavorable if the status of patient was dead.

**Table 1:** Summary of the preoperative radiological classification.

Preoperative radiological parameters									
Hyperintense signal on DWI		Heterogeneous enhancement on T1-weighted Gd-enhanced MRI		Disappeared/ Disintegrated arachnoid layer on T2-weighted MRI		Presence of PTE on T2-weighted MRI		Irregular tumor shape	
No	Yes	No	Yes	Intact	Yes	No	Yes	No	Yes
0	1	0	1	0	1	0	1	0	1

Scoring*	0	1	2	3	4	5
Groups^#^	I		II		III	

*Each “Yes” scores 1 point; the lowest possible score is 0 and the highest is 5.

^#^ Group I—score 0–1; group II-score 2–3; group III—score 4–5.

### Statistical analysis

The continuous variables were represented as mean ±SD. Univariate analyses were conducted to examine the association between radiological and histopathological features. Multivariate logistic regression model was used to evaluate if the radiological factors predict the occurrence of non-grade I meningioma (grade II and grade III). Kaplan-Meier analysis was utilized to evaluate the progression free survival and overall survival time and rate. Cox regression analysis model was used to determine the predictors of prognosis. The independent variable included; age at the time of surgery, gender, WHO-grading, preoperative radiological parameters and the radiological classification groups. Kappa test was used to study the accuracy of association between histopathological grading and proposed radiological scoring. For all analysis, p < 0.05 were considered statistically significant. SPSS 20 (Chicago, Inc, IBM) was used for all the statistical analysis.

## Results

The mean age of 101 men and 145 women was 57.5 years (range, 7–80 years).

### MRI findings and scoring

Thirty-three percent patients (82/246) demonstrated hyperintensity on DWI image, 32% (79/246) of patients showed heterogeneity on T1-weighted gadolinium enhanced MRI; 41% (100/246) had disruption of arachnoid on T2-weighted MRI, 39% (97/246) demonstrated PTE on T2-weighted MRI, and 24% (59/246) patients had irregular shape of tumor. Scoring for preoperative radiological features revealed, 17.4% (43/246) of the patients had preoperative radiological score of 0, 36% (88/246) had score 1, 21% (52/246) score 2, 14% (35/246) score 3, 8% (20/246) score 4, and 3% (8/246) had score 5. Based on this, 53% (131/246) of patients were classified as group I, 35% (87/246) group II and 11% (28/246) were group III.

### Histopathological classification

Based on WHO 2007 histopathological classification system, 76% (187/246) of the patients were grade I, 15% (37/246) were grade II and 9% (22/246) were grade III. For data analysis, WHO grade II and III patients were defined as non-grade I tumors, 24% (59/246) of the patients. Seventy percent (131/187) of patients with grade I tumors were classified as group I, 30% (56/187) were group II, and 0% were group III. For non-grade I tumors 0% of patients were group I, 54.2% (32/59) were group II and 47.4% (28/59) were group III. There was a significant correlation between the radiological groups and histopathological grades of meningioma (Pearson Chi-square-136.2, P< 0.0001). Among Group II (87) cases, 64% (56/87) of patients were WHO grade-I and 36% (31/87) were non-grade I tumors.

All the five preoperative radiological scoring parameters were significantly associated with non-grade I tumor, controlling for age and gender (Table 2). Hyperintensity on DWI was the strongest independent predictor of non-grade I meningioma (P<0.001, OR-17, CI-5.8–47.6), followed by disruption of arachnoid layer (P<0.001, OR-14, CI-4.3–42.3), PTE on T2-weighted MRI (P<0.001, OR-9, CI-3.1–26.8), heterogenicitiy on T1-weighted gadolinium enhanced MRI (P<0.001, OR-6.1, CI-2.2–17.1) and irregular shape of the tumor (P<0.001, OR-6.1, CI-2.1–17.5) (Table 2). The preoperative radiological scoring system demonstrated moderate accuracy (Kappa value, 0.511; p<0.001).

**Table 2:** Logistic Regression analysis for the clinical and radiological parameters as predictors of WHO non-grade I meningioma.

Parameters	OR	95% CL for OR		P-value
		Lower	Upper	
dwi	16.659	5.823	47.661	<0.001
T1gd	6.160	2.218	17.108	<0.001
AR	13.567	4.349	42.324	<0.001
PTE	9.094	3.079	26.863	<0.001
Shape	6.182	2.175	17.572	<0.001
Gender	1.042	.371	2.924	.938
Age	.999	.971	1.029	.967

### Overall survival

**Fig 2:**
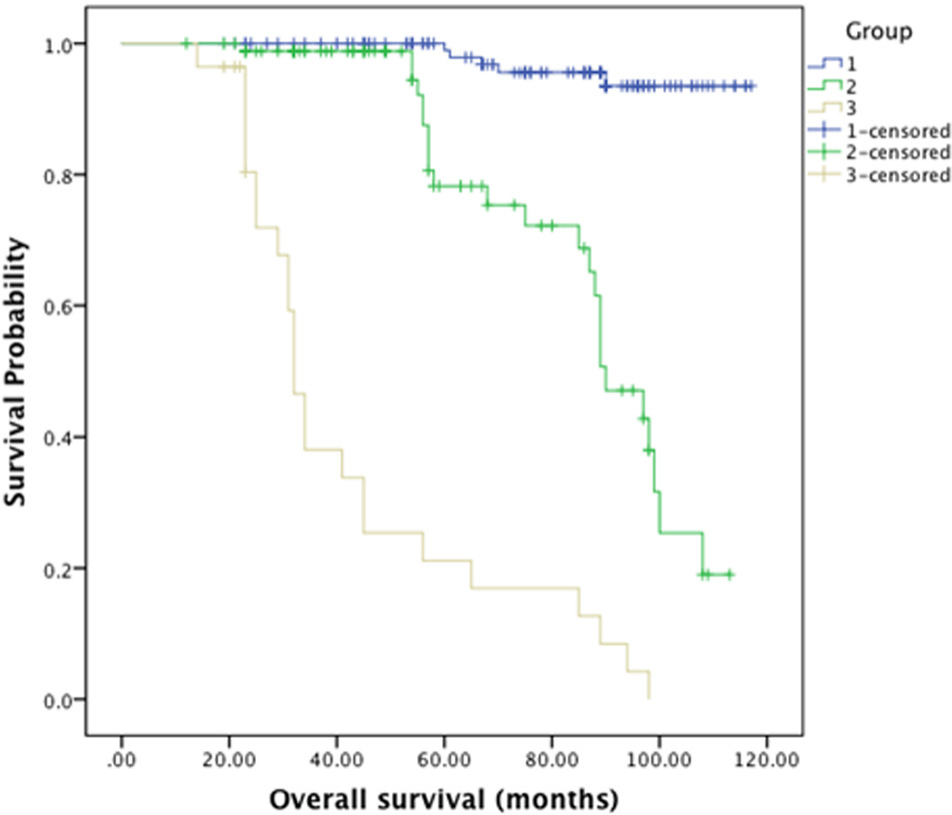
Kaplan-Meier Curve for Survival analysis among the group I, II and III.

**Fig 3:**
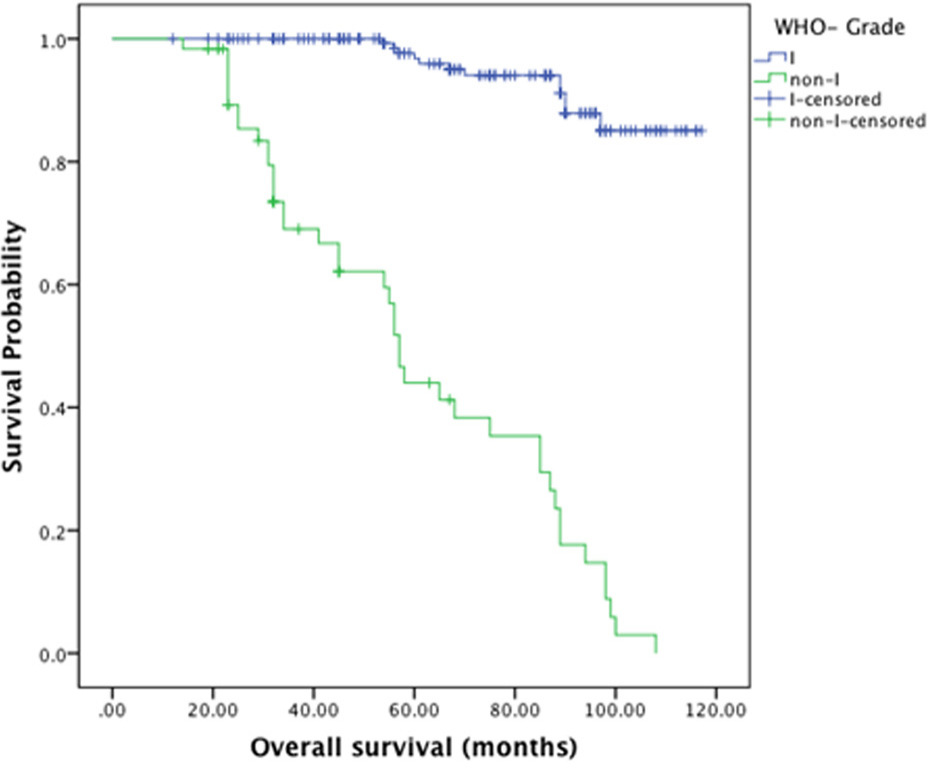
Kaplan-Meier Curve for Survival analysis for WHO grade I and non-grade I tumors.

**Fig 4:**
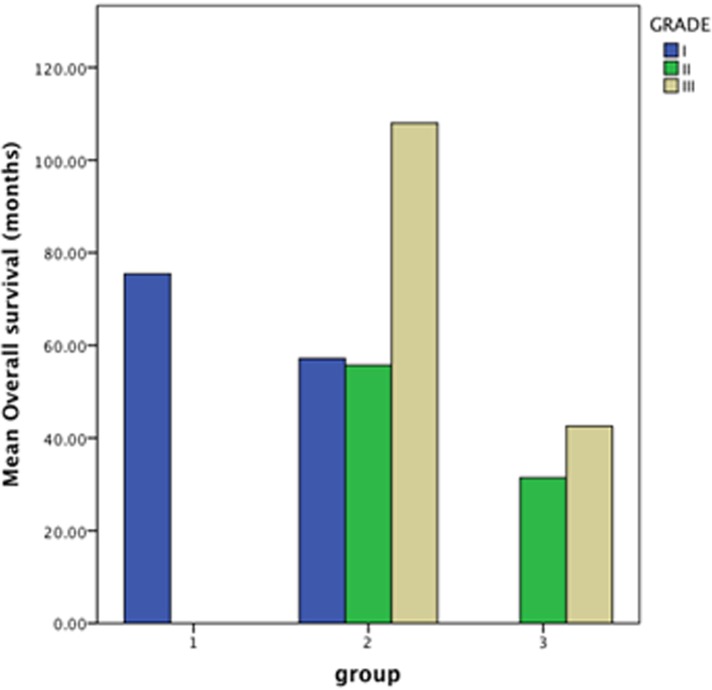
Bar-graph demonstrating the survival time in group I, II and III in relation to WHO grades of meningioma.

The mean follow-up period was 94.6 months (range 12–117 months; median 80.4 months). The median overall survival was 97.5 ± 2.2 months. The median survival in patients with WHO grade I tumors was 111.4 ± 1.5 months and 60.7 ±4.1 months in patients with WHO non-grade I tumors (Fig. 2). For patients in groups I, II and III, the median survival was 114.1±1.2, 88± 3.3, 43.2± 5.1 months respectively (Fig. 3).The overall survival rate was 78.5%; the patients in groups I, II and III had the survival rates of 96.2%, 72.4% and 14.3% respectively. Fig. 4 demonstrates the survival relationship among various groups and grades of meningioma. In cox regression analysis model, age at the time of surgery (P<0.0001, OR–1.039, CI—1.017–1.062), WHO-grade (P = 0.017, OR—3.014, CI—1.217–7.465), and preoperative radiological classification groups II (P = 0.002, OR—6.194, CI—1.956–19.610) and group III (P<0.0001, OR—21.658, CI—5.701–82.273) were independent predictors of unfavorable overall survival outcomes (Table 3).

**Table 3:** Cox Regression analysis for the clinical and radiological parameters as predictors of unfavorable survival outcomes.

Parameters	OR	95% CL for OR		P-value
		Lower	Upper	
age	1.039	1.017	1.062	<0.001
WHO-grade	3.014	1.217	7.465	0.017
Radio-Group II	6.194	1.956	19.610	0.002
Radio-Group III	21.658	5.701	82.273	<0.001

### Progression free survival-

**Fig 5:**
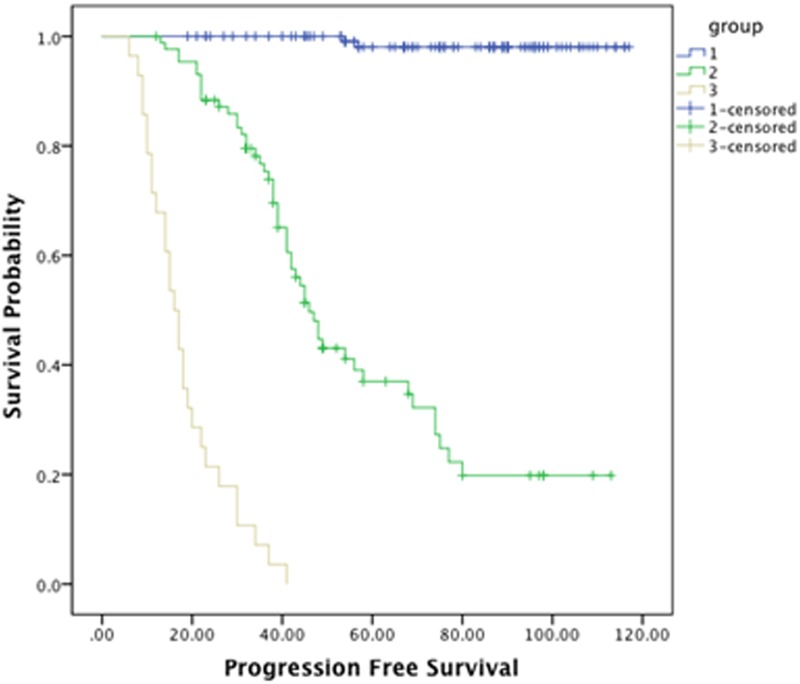
Progression free survival analysis among the groups I, II and III.

Fig. 5 demonstrates PFS in groups I, II and III. The median PFS was 86.2 ± 2.7 months and the PFS rate was 67%. The median PFS for patients in groups I, II and III was 115.7± 0.8, 58.5±3.9, 18.2±1.7 months and the PFS rate was 98.5%, 42% and 0% respectively. The median PFS rate and time for patients with grade I tumors was 104.5±2.1 and 86% months and 27.5 ± 2.1 months and 10% for non-grade I tumors. The rate of recurrence was 100% in group III patients and 1.5% and 59% in groups-I and II respectively. In cox regression analyses, the WHO grade (P<0.001, OR–4.712, CI—2.574–8.627), group II (P<0.001, OR–52.504, CI–12.367–222.9) and group III (P<0.0001, OR–249.22, CI—51.822–1198.561) were predictors of recurrence (Table 4).

**Table 4:** Cox Regression analysis for the clinical and radiological parameters as predictors of recurrence.

Parameters	OR	95% CL for OR		P-value
		Lower	Upper	
WHO-grade	4.712	2.574	8.627	<0.001
Radio-Group II	52.504	12.367	222.9	<0.001
Radio-Group III	249.22	51.822	1198.561	<0.001

## Discussion

In present study, hyperintensity on DWI-MRI, disruption of arachnoid ring on T2-weighted MRI, PTE on T2-weighted MRI, heterogeneity on T1-weighted gadolinium enhanced MRI, and irregular shape of tumor were all significant predictor of WHO non-grade I meningioma. Among the five parameters, hyperintensity on DWI was the strongest independent predictor of non-grade I meningioma in our study. Several authors have demonstrated that hyperintensity on DWI and decreased co-efficient on ADC values predicts the higher histological grades of meningioma [16,24,25,34]. However, others have demonstrated that DWI cannot accurately predict the histopathological grading of meningioma [36,37]. Based on the hypothesis, that the diffusion of water to and from the cells is highly dependent on the ratio of intracellular and extracellular space, DWI is used to differentiate the tumor grades [3,21,5,31]. High grade meningiomas are characterized by increased tumor cellularity, increased nucleus/cytoplasmic ratio, small cell size, and increased mitotic cells, which restricts the water diffusion; depicting as hyperintensity on DWI [25,32–35]. Another pathological feature associated with aggressive meningioma behavior is disruption of the brain-tumor interface [17,20,21].The presence of arachnoid ring on T2-weighted MRI indicates a clear brain-tumor interface[38]. The high-grade meningioma, can penetrate into the brain parenchyma by direct invasion of the tumor cells into the neurovascular tissue [39]. The slow growing tumor, however, penetrates the sub-pial tissue and causes absorption of arachnoid membrane resulting in disruption of the brain-tumor interface. Thus, disruption of the hyperintense arachnoid ring on the T2-weighted MRI represents not only histopathological aggressiveness but also the invasive nature of otherwise benign grade I meningiomas.

The signal intensity on T2-weighted MRI is associated with the amount of PTE and histological type of meningioma [10,15,28,29].Surgically, the presence of PTE might indicate a more difficult tumor resection, aggressive meningioma and disruption of arachnoid layer at the tumor brain-interface [23,40]. PTE on the MRI, however, has been attributed to several other factors including tumor size [40,41], location [17,42,43], histological grading [17,19,44], tumor vascularity [19,29,40,45], tumor-related venous obstruction [29,46],impairment of blood-brain barrier [2,20,23,29,46],presence of pial-cortical blood supply [29,47],vascular endothelial growth factor [45] and irregular tumor margin [29,41].Thus the presence of PTE alone might not accurately predict the aggressiveness of meningioma. Similarly, heterogeneous enhancement on MRI is another feature associated with high-grade meningioma. It indicates intra-tumoral necrosis and heterogeneous distribution of the proliferating cells [2,6,7]. Some WHO- grade I meningiomas with large size, calcification or cystic degeneration, however, also demonstrates heterogeneous enhancement on MRI. Thus, the sole presence of heterogeneous enhancement on MRI might not accurately predict the aggressive meningioma behavior. Traditionally, the irregular tumor shape, is associated with high-grade meningiomas. The WHO grade-I meningiomas usually are globular shape [2,8,29]; however, in instances where the vascular supply to the tumor is impeded, the part of tumor deprived of blood supply undergoes necrosis and dies. This can cause a lobulated appearance of the otherwise benign tumor. Thus, the shape of tumor alone might be a disguise for meningioma aggressiveness.

The proposed preoperative radiological classification predicted the aggressive histopathological, and survival outcomes in patients with convexity meningiomas. There was a moderately accurate association between the preoperative scoring system and WHO histopathological classification system. The patients with group II and III were more likely to have unfavorable survival outcome compared to patients with group I. Most WHO grade I patients were classified as group I and had favorable survival outcomes, however, 30% (56/187) of WHO-grade I tumors were classified as group II. The overall survival was significantly higher in patients with histopathological grade III and preoperative radiological classification group II meningioma compared to grade III and group III meningiomas. Similarly, the patients with grade I and group I had higher survival rates compared to grade I and group II. Therefore, there exist a group of grade I meningioma that have aggressive radiological features and clinical behavior; however, any of this five preoperative MRI parameters alone might not accurately predict the aggressive clinical behavior of all grade I and non-grade I meningiomas. The preoperative radiological classification, presented in the current study, can be used as a supplement to the WHO histopathological meningioma grading, to accurately predict the aggressive behavior of convexity meningioma.

### Study limitations

Despite the contributions this study makes to the literature, there are several limitations to this study. First, the study suffers from the inherent bias introduced by its retrospective study design. Second, the study is underpowered due to relatively small number of patients in groups II and III. Further studies are needed to validate this classification, which will then be able to define the need for additional treatment including postoperative radiotherapy or chemotherapy and the need for shorter follow-up in patients with groups II and III convexity meningioma.

## Conclusion

We introduce a preoperative radiologic classification to predict the radiological and clinical aggressive behavior of convexity meningioma. Group I meningioma demonstrated benign radiologic, histopathologic and clinical behavior; group III demonstrated aggressive radiologic, histopathologic and clinical behavior. Group II meningioma might be considered intermediate as some histopathologically benign tumors belonged to group II; the need for more aggressive follow-up and/or treatment should be further investigated.
